# Supplementary material for: Burkholderia collagen-like protein 8, Bucl8, is a unique outer membrane component of a putative tetrapartite efflux pump in Burkholderia pseudomallei and Burkholderia mallei
Source: PLoS One. 2020 Nov 23;15(11):e0242593. doi: 10.1371/journal.pone.0242593 (PMC7682875; doi:10.1371/journal.pone.0242593)
Supplement: S1 Table — (DOCX) [file pone.0242593.s001.docx]

S1 Table. Primers

| **Target** | **Primer name** | **Primer sequence** |
| --- | --- | --- |
| *fusR* | pSL522-ApaI-F | 5’-GAAGGGCCCATGCTTGCGCATCCGG-3’ |
|  | pSL522-HindIII-R | 5’-GGTAAGCTTCGGGCATCACGCGCACG-3’ |
|  | BurkhLysR-2F | 5’-GTTCGTCCGCGTGGTCGATG-3’ |
|  | BurkhLysR-2R | 5’-CGCAAGTGCGCCTCGAGATC-3’ |
| *bucl8* | Bucl8-1F | 5’-CTCGTATGAAGAGGCGATCC-3’ |
|  | Bucl8-3F | 5’-CTACGCGCTCCTCGACATCGCGC-3’ |
|  | Bucl8-3R | 5’-TGCGTGCCGATGCCCGCGCGCA-3’ |
| *fusCD* | BurkhFusBCD-1F | 5’-GTGGCTCTATCTCGCGAAGGCGC-3’ |
|  | BurkhFusBCD-1R | 5’-GCGGCTGCATCACGATGAACACGG-3’ |
| *fusE* | BurkhFusE-1F | 5’-CAGCCGTCATCCTGATCGTCGCG-3’ |
|  | BurkhFusE-1R | 5’-CGGCGCGACGTTGACGATCTCC-3’ |
| *tar* | pSL523-ApaI-2F | 5’-CGACTTGCGCTTGCCGCCGGGCCCTTG-3’ |
|  | pSL523-ApaI-2R | 5’-GAAGGGCCCGCGACGAGCATGGGGCAAC-3’ |
|  | BurkhTar-1F | 5’-CGCACGATGGACGAGGTCGTGC-3’ |
|  | BurkhTar-1R | 5’-CCCGCGCTCTGCTCACTCGACG-3’ |
| Plasmids | pMo130-MCSI-F | 5’-GCTCACATGTTCTTTCCTGCG-3’ |
|  | pMo130-MCSI-R | 5’-CCCGGTCGCATTACACCTTTG-3’ |
|  | pSL520-F | 5’-CACGGATCCTCGACTGC-3’ |
|  | pSL520-R | 5’-CAAGCTTTAGCGAGCTGCA-3’ |
|  | pSL521_1F | 5’-GAGGAGAAATTAACTATGAGAGGATCG-3’ |
|  | pSL521_2R | 5’-AGCTAATTAAGCTTTAGCGAGCTG-3’ |
|  | pQE30-F | 5’-CACCTGACGTCTAAGAAACCATTAT-3’ |
|  | pQE30-2R | 5’-TCTCCATTTTAGCTTCCTTAGCTCC-3’ |
| FUSC | FusC 2-F | 5’-TGTCGCTCATCGTCGTCTA-3’ |
|  | FusC 2-R | 5’-AGCGGCGTGAATTTCTCTT-3’ |
|  | FusC 3-F | 5’-GATCGTGACGGCGATCATCTG-3’ |
|  | FusC 3-R | 5’-GGAAACAGCAGCACGATTGTC-3’ |
|  | FusC 4-2F | 5’-CCACGGCGATAACGAGATCGC-3’ |
|  | FusC 4-2R | 5’-CACGAGTAGGTCGCATACAGC-3’ |
|  | FusC 5-2F | 5’-CGATGAGCGGGATGGTGCTC-3’ |
|  | FusC 5-2R | 5’-CGCGACCCACAGCGTGATGC-3’ |
|  | FusC 6-F | 5’-CGTCGATCGCGACGGTATCG-3’ |
|  | FusC 6-R | 5’-GCACGACAGCGACAGAAAGCC-3’ |
| 16s | 16s rRNA-F | 5’- GGCTAATACCCGGAGTGGA-3’ |
|  | 16s rRNA-R | 5’- CTAGCCTGCCAGTCACCAA-3’ |
