# Supplementary material for: Burkholderia collagen-like protein 8, Bucl8, is a unique outer membrane component of a putative tetrapartite efflux pump in Burkholderia pseudomallei and Burkholderia mallei
Source: PLoS One. 2020 Nov 23;15(11):e0242593. doi: 10.1371/journal.pone.0242593 (PMC7682875; doi:10.1371/journal.pone.0242593)
Supplement: S2 Table — (DOCX) [file pone.0242593.s002.docx]

S2 Table. Genes and associated identification numbers of FusC loci

|  | **Bp 1026b** | | **Bp K96243** | | **Bm ATTC 23344** | |
| --- | --- | --- | --- | --- | --- | --- |
| **Gene** | **Locus tag** | **Protein ID** | **Locus tag** | **Protein ID** | **Locus tag** | **Protein ID** |
| *fusC 2* | BP1026B_RS11380 | [WP_004552879.1](https://www.ncbi.nlm.nih.gov/protein/490656881) | BPS_RS06755 | [WP_004534567.1](https://www.ncbi.nlm.nih.gov/protein/490669577) | DM55_RS13405 | [WP_004193403.1](https://www.ncbi.nlm.nih.gov/protein/490297964) |
| *fusC 3* | BP1026B_RS12100 | [WP_004198110.1](https://www.ncbi.nlm.nih.gov/protein/490302733) | BPS_RS05900 | [WP_004198110.1](https://www.ncbi.nlm.nih.gov/protein/490302733) | DM55_RS12800 | [WP_004198110.1](https://www.ncbi.nlm.nih.gov/protein/490302733) |
| *fusC 4* | BP1026B_RS21370 | [WP_004187681.1](https://www.ncbi.nlm.nih.gov/protein/490292089) | BPS_RS21435 | [WP_004187681.1](https://www.ncbi.nlm.nih.gov/protein/490292089) | DM55_RS20330 | [WP_004202241.1](https://www.ncbi.nlm.nih.gov/protein/490307172) |
| *fusC 5* | BP1026B_RS22725 | [WP_004539022.1](https://www.ncbi.nlm.nih.gov/protein/490674193) | BPS_RS22800 | [WP_004539022.1](https://www.ncbi.nlm.nih.gov/protein/490674193) | DM55_RS24535 | [WP_004195004.1](https://www.ncbi.nlm.nih.gov/protein/490299590) |
| *fusC 6* | BP1026B_RS28905 | [WP_004552079.1](https://www.ncbi.nlm.nih.gov/protein/490687587) | BPS_RS29185 | [WP_004525018.1](https://www.ncbi.nlm.nih.gov/protein/490660028) | DM55_RS17915 | [WP_004190560.1](https://www.ncbi.nlm.nih.gov/protein/490295011) |

Data were retrieved from NCBI for *B. pseudomallei* 1026b, *B. pseudomallei* K96243, and *B. mallei* ATTC 23344 reference genomes. Proteins were labeled as FUSC family protein.
