# Supplementary material for: Burkholderia collagen-like protein 8, Bucl8, is a unique outer membrane component of a putative tetrapartite efflux pump in Burkholderia pseudomallei and Burkholderia mallei
Source: PLoS One. 2020 Nov 23;15(11):e0242593. doi: 10.1371/journal.pone.0242593 (PMC7682875; doi:10.1371/journal.pone.0242593)
Supplement: S3 Table — (DOCX) [file pone.0242593.s003.docx]

**S3 Table. gBlock inserts for construction of recombinant proteins**

| Construct/ protein | Amino Acid Sequence | Nucleotide Sequence |
| --- | --- | --- |
| pSL520/ rBucl8-Ct | MRGSHHHHHHGSSTAGASATASASAAGHAPAGAAAPASPAGIRAAASARASMPAPAAAATAPAFASPVAGASTPMPAATAAARAAR | CACGGATCCTCGACTGCGGGAGCATCTGCAACTGCAAGCGCAAGCGCGGCTGGCCATGCACCGGCTGGCGCGGCAGCTCCAGCGTCACCAGCTGGCATTCGCGCTGCTGCTTCGGCGAGAGCGTCTATGCCGGCACCAGCCGCAGCGGCAACTGCGCCTGCCTTTGCATCACCGGTGGCTGGTGCTTCGACTCCGATGCCTGCAGCAACCGCAGCTGCTCGTGCAGCTCGCTAAAGCTTG |
| pSL521/ rBucl8-CL-Ct | MRGSHHHHHHGSGASGASGASGASGASGASGASGASGASGASGASGASGASGASGASGASGASGASSTAGASATASASAAGHAPAGAAAPASPAGIRAAASARASMPAPAAAATAPAFASPVAGASTPMPAATAAARAAR | GAGGAGAAATTAACTATGAGAGGATCGCATCACCATCACCATCACGGATCCGGTGCTTCGGGTGCTTCGGGTGCCTCGGGTGCCTCGGGTGCCTCGGGTGCCTCGGGTGCCTCGGGTGCCTCGGGTGCCTCGGGTGCCTCGGGTGCCTCGGGTGCCTCGGGTGCCTCGGGTGCCTCGGGTGCCTCGGGTGCCTCGGGTGCCTCGGGTGCCTCGGGTGCCTCGGGTGCCTCGGGTGCTTCGTCGACTGCGGGAGCATCTGCAACTGCAAGCGCAAGCGCGGCTGGCCATGCACCGGCTGGCGCGGCAGCTCCAGCGTCACCAGCTGGCATTCGCGCTGCTGCTTCGGCGAGAGCGTCTATGCCGGCACCAGCCGCAGCGGCAACTGCGCCTGCCTTTGCATCACCGGTGGCTGGTGCTTCGACTCCGATGCCTGCAGCAACCGCAGCTGCTCGTGCAGCTCGCTAAAAGCTTAATTAG |
