# Supplementary material for: Burkholderia collagen-like protein 8, Bucl8, is a unique outer membrane component of a putative tetrapartite efflux pump in Burkholderia pseudomallei and Burkholderia mallei
Source: PLoS One. 2020 Nov 23;15(11):e0242593. doi: 10.1371/journal.pone.0242593 (PMC7682875; doi:10.1371/journal.pone.0242593)
Supplement: S1 Raw image — (PDF) [file pone.0242593.s005.pdf]

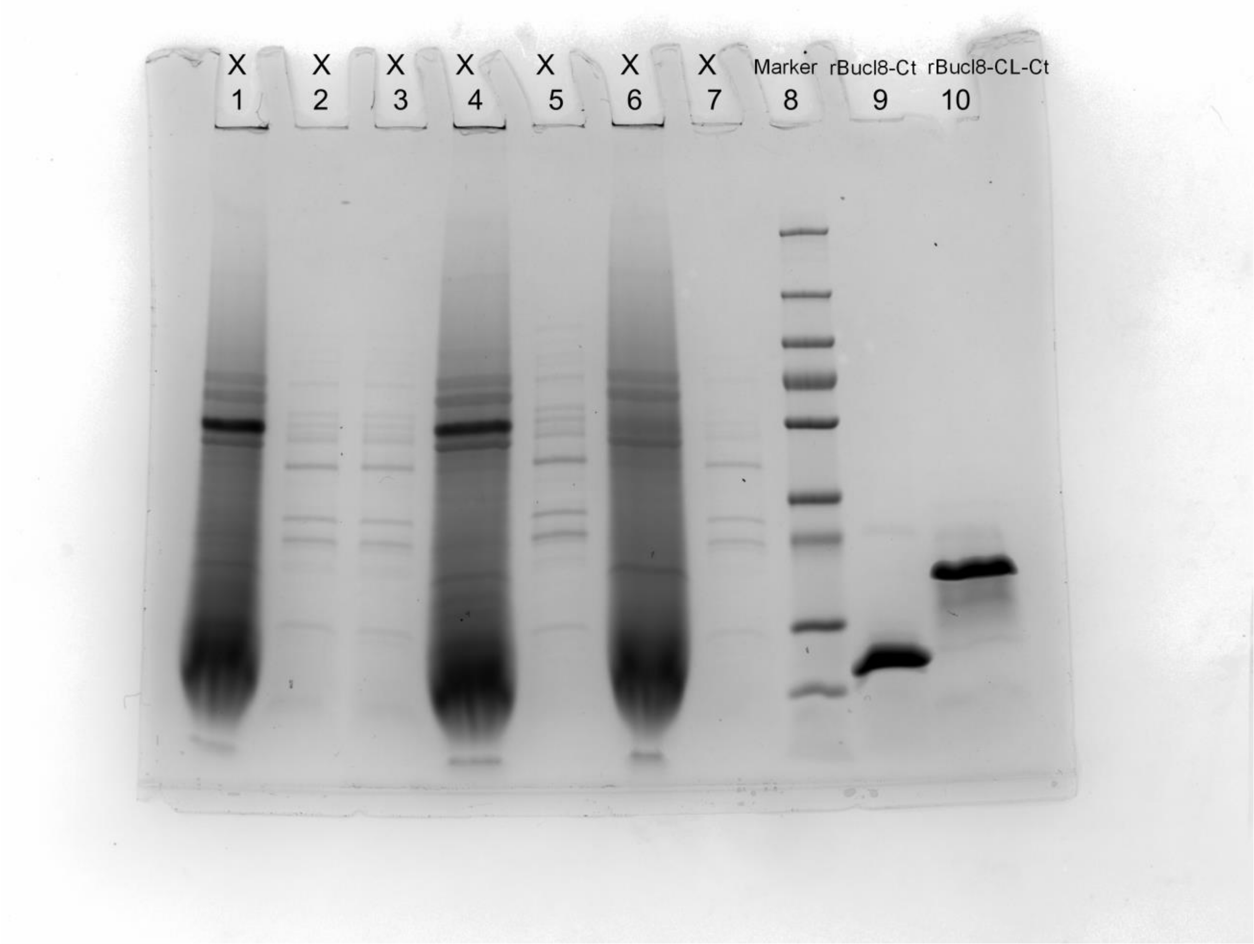

S1 raw image. Uncropped and unadjusted SDS-PAGE analysis of purified rBucl8-Ct and rBucl8-CL-Ct constructs from Fig 1D.

3 µg of recombinant proteins rBucl8-Ct (Lane 9) and rBucl8-CL-Ct (Lane 10) were resolved on a 4-20% SDS-PAGE (BioRad Mini-PROTEAN TGX). Lanes 1 thru 7 are samples unrelated to this publication (indicated by X above lane number). Lane 8 is the marker (Thermo; Page Ruler Plus Prestained Protein Ladder) with following molecular sizes (kDa) from bottom to top: 10, 15, 25, 35, 55, 70, 100, 130, 250.
